# The Level of Knowledge, Attitude and Practice of the Physicians and Nurses About Suitable Healthcare Personnel (HCP) Attire in Hospitals of Tehran University of Medical Sciences 

**Published:** 2019-03

**Authors:** Jayran Zebardast, Nikzad Eisazadeh, Khorshid Vaskooi, Fatemeh Mirbazegh

**Affiliations:** 1 Institute for Cognitive Science Studies (ICSS), Tehran, Iran; 2 Quran, Hadith and Medicine Research Center, Tehran University of Medical Sciences, Tehran, Iran; 3 Treatment Vice Chancellor, Tehran University of Medical Sciences, Tehran, Iran; 4 Treatment Vice Chancellor, Imam Khomeini Hospital, Tehran University of Medical Sciences, Tehran, Iran

**Keywords:** Knowledge-Attitude, Performance, physicians, Nurses, Professional Attire

## Abstract

**Objective:** The purpose of this study was to investigate the status of knowledge, attitude and practice of medical team about suitable " Healthcare personnel (HCP) attire".

**Materials and methods:** This is a descriptive study that was approved by the Research Ethics Committee of Tehran University of Medical Sciences, and evaluated knowledge, attitude and performance of physicians and nurses about "Health care personnel (HCP) attire" by a questionnaire. In order to create the questionnaire a panel of experts’ reviews was set and a questionnaire was made through Focus group discussion (FGD). The Variables included age, gender, work experience, type of employees' time, type of jobs, education level, type of employee.

**Results:** This study was conducted on 441 physicians and nurses who were working in Tehran University of Medical Sciences. The mean percent of KAP score was 72.6 ± 14.3. The score of the questionnaire in general was 14.91 ± 70.99 for knowledge, 73.5 ± 13.3 for attitude and 73.7 ± 17.1 for performance.

**Conclusion:** According to this survey, the questionnaire score in the general knowledge, attitude and performance about the "Healthcare personnel (HCP) attire" is low.

## Introduction

As traditionally, professional' attire is an important of the physicians and nurses in culture. Professional' attire is the first impression for the medical team. The purposes of Professional' dress is neatness, cleanliness, identification, and also make-up, hairstyle, facial expressions (as a part of a suitable professional) have roles in public verbal and non-verbal communication. Sociologists and psychiatrists have the great emphasized influence on a positive relationship by professional ' dress, and show the style and wearers' confidence (1-6). The reflection importance of covering the physicians in the relationship between a doctor and a patient can also see in Hippocrates's words, which sentences about professional ethics and their attire (6-12). On the other hand, Physicians and nurses are probably at risk in exposure to a great contaminated with the range of pathogens through contact with infectious body fluids, injuries, blood and mucous membrane extraction, and another source of infections. So they should use the best dress to protect themselves from these factors (13-15). California by Damon E. Anastacia (16), Clavelle Joanne T., Goodwin Miki, Tivis Laura J. (17), in their studies show that more respect and attention can attract by the medical team to their clothes. Despite this, it seems that lack of evidence that regard to the effect of Physicians and nurses ' Knowledge, Attitude and Performance bout suitable dress characteristics that can be affect from some criteria. Finding the lack of such studies, we decided to evaluated attitude and performance of the physicians and nurses about their "Healthcare personnel (HCP) attire" in Tehran University of Medical Sciences of Iran.

## Materials and methods

This is a descriptive study that approved by the Research Ethics Committee of Tehran University of Medical Sciences, and evaluated knowledge, attitude and performance of physicians and nurses about "Healthcare personnel (HCP) attire" by a questionnaire. (Registration number: IR.TUMS.REC.1394.2184). 

Informed consent was taken from all the participants. In order to create the questionnaire panel of expert's reviews was set and a questionnaire through Focus group discussion (FGD) was made. The questionnaire was evaluated by qualitative and quantitative methods (so Cranach's alpha test as fair (0.8) reported and the reliability (questions correlation) was calculated near 92%). For domain items of "HCP attire", some articles were reviewed and consulted. 15 items that divided into 3 domains with closed questions was created. This questionnaire had 15 questions (5 questions in each knowledge, attitude and performance). The Likert scale used for the answer options and maximum score was set to be 5 and total score of the questionnaire measured from100 scale. All participants filled this questionnaire. The variables included age, gender, and work experience, ward, type of employees' time, type of jobs and education level asked, too. Data were analyzed using by using statistical package of social science Software (SPSS version 16, Chicago, IL, USA). T-Test and ANOVA used for quantitative variables and chi-square for qualitative variables, too. The significance level considered as < 0.05.

## Results

In this study, 441 physicians and nurses working in Tehran University of Medical Sciences studied. The demographic characteristics of people listed in table 1.

**Table 1:** Demographic characteristics of participants

		**Frequency**	**Percent**
Sex	Male	344	78
	Female	97	22
Age	<35	296	67/1
	>35	145	32/9
Work experience	<10	360	81/6
	>10	81	18/4
Type of employees' time	Full time	384	87/1
	Half time	57	13/9
Type of jobs	Nurse	301	68/25
	physician	140	31/746
Education level	Upper that diploma	9	2
	Bachelor	270	61/2
	Master	22	5
	PhD and upper	140	31/7
Type	Cadets course	25	5/7
	Conventional	138	31/3
	Official	67	15/2
	Contractor	211	47/8
Ward	Internal departments	164	37/2
	Emergency departments	94	21/3
	surgery rooms	65	14/7
	Clinics	95	21/5
	ICUs	23	5/2
	Total sum	441	100

**Table 2:** Participants' Knowledge, Attitude and performance according to their demographic characteristics

		**Knowledge**	**Attitude**	**performance**
Sex	P value	0.6	0.7	0.1
	< 35	71.01 ± 14.46	73.52 ± 13.17	74.59 ± 17.22
	> 35	71.77 ± 14.5	73.13 ± 13.26	72.05 ± 16.13
Age	P value	0.5	0.6	0.2
	Male	71.50 ± 14.76	73.45 ± 13.09	73.57 ± 17.05
	Female	70.43 ± 13.44	73.20 ± 13.59	74.43 ± 16.42
Type of jobs	P value	0.02	0.5	0.3
	Nurse	70.94 ± 14.31	73.42 ± 13.11	73.67 ± 16.67
	Physician	77.45 ± 16.31	72.91 ± 15.01	75.45 ± 21.18
Type of employees' time	P value	0.049	0.04	0.09
	Full time	70.66 ± 14.44	72.96 ± 13.03	73.86 ± 15.40
	Half time	74.06 ± 12.63	78.51 ± 13.09	71.54 ± 27.23
Education level	P value	0.03	0.09	0.09
	Upper that diploma	70.64±14.89	73.59 ± 13.59	75.41 ± 15.91
	Bachelor	77.45 ± 16.31	72.91 ± 15.01	75.45 ± 21.18
	Master	70.63 ± 13.02	73.34 ± 11.69	70.00 ± 17.48
	PhD and doctor specialist	84.89 ± 8.89	69.78 ± 19.40	78.67 ± 18.44
Type	P value	0.05	0.40	0.001
	Cadets course	70.09 ± 13.37	72.35 ± 11.04	79.97 ± 11.00
	Conventional	75.29 ± 11.26	71.11 ± 13.77	60.09 ± 20.53
	Official	71.04 ± 12.18	74.08 ± 12.39	95.68 ± 1.11
	Contractor	70.56 ± 15.59	74.54 ± 14.21	69.84 ± 15.74
Ward	P value	0.10	0.05	0.70
	Internal departments	71.78 ± 14.39	73.95 ± 11.29	73.68 ± 16.91
	Emergency departments	71.62 ± 14.75	72.68 ± 14.18	74.60 ± 15.96
	surgery rooms	68.06 ± 12.78	69.72 ± 13.69	71.57 ± 16.97
	Clinics	71.03 ± 15.28	74.95 ± 14.27	75.07 ± 16.73
	ICUs	76.17 ± 14.19	76.35 ± 14.32	71.65 ± 21.23
Work experience	P value	0.20	0.10	0.50
	< 10	70.77 ± 14.93	73.23 ± 13.59	73.88 ± 7.15
	> 10	74.15 ± 14.54	77.23 ± 9.78	72.00 ± 17.05

The score of the questionnaire in the general 14.91 ± 70.99 was for knowledge, 73.5 ± 13.3 was for attitude 73.7 ± 17.1 was for performance. Participants' knowledge, attitude and performance according their demographic characteristics were shown in table 2.

## Discussion

Professionalism and infection control had different aspects of "professional attire" (18, 19). In article by Brandt et al it be found that today young Physicians and nurses students may choose informal rather than casual attire, so, in the New Millennium the Old Dress Codes' Values is more concerned and "professional attire" is known as a measure of truth for our patients comforted feels' but obviously physician' team members may not aware about ignoring Hippocrates' advice that so he said that" they should be well-dressed, anointed sweet smelling and so clean in person"(20). In a study by Gjerdingen et al. (9) it is founded that physician's appearance through questionnaires were evaluated from all physicians during 3 residency programs. The name tag, shirt and dress pants white coat, dress shoes, nylons skirt or dress is the most important things that them had positive reaction to them as traditional physician attire but some Negative responses were seen about scrub suits, clogs, sandals, blue jeans, athletic shoes) and also, more older than did younger participants had favored  traditional and professional attire. It became clear that the type of "Healthcare personnel (HCP) attire "has a great influence on treatment confidence but in other hands, it is more neglected the knowledge and attitude of "Physicians and nurses "in hospitals about their Clothing. In this study, 441 physicians and nurses working in Tehran University of Medical Sciences studied, according to our result the mean percent of KAP score was 72.6 ± 14.3, so, this shown, Physicians and nurses had moderate knowledge, attitude level and performance respectively and maybe it's be as a problem in protecting themselves from pathogen agents (13-15) or perhaps they cannot have good communication with their patents (21-23).

In sum up, it seems that today there is a decline in formal attire among Healthcare members but patients still prefer a formal dress than informal appearance for Healthcare personnel, so it is a concern by medical members staff in their opinions about the formal dress, and they should be more attention about their attire than their patients (24-26). 

## Conclusion

According to this survey, it can successfully establish that "Healthcare personnel (HCP) attire" is part of the treatment approach and so, It should be considered and to extend research in this area. So, some educational programs with the focus on ethical approaches and with consideration of standard precautions are recommended and also it is important to change dressing medical healthcare staffs to more suitable dress this may affect on therapeutic doctor-patient interaction, too.
